# Low Incidence of Miscarriage Induced by the Scent of Male Littermates of Original Mates: Male Kinship Reduces the Bruce Effect in Female Mice, *Mus musculus*

**DOI:** 10.1371/journal.pone.0068673

**Published:** 2013-07-17

**Authors:** Yuting Wang, Dingzhen Liu

**Affiliations:** Ministry of Education, Key Laboratory of Biodiversity Science and Ecological Engineering, College of Life Sciences, Beijing Normal University, Beijing, China; Utrecht University, The Netherlands

## Abstract

The scent of a novel male can elicit pregnancy block in recently mated female mice (*Mus musculus*), a phenomenon known as the Bruce effect. Despite abundant literature on the Bruce effect in rodents, it remains unclear whether males related to a female’s original mate can induce the Bruce effect in out-bred, communally living mice. We investigated this question using Kunming (KM) male mice of varying genetic relatedness. Recently mated females were subjected to three treatments: exposure to the urine of the mate, urine of the mate’s male littermate, and urine of a male unrelated to the mate. It was found that the urine of male littermates of the females’ mates did not elicit more pregnancy block than that of the females’ mates. However, the urine of novel males caused a higher rate of female miscarriage than that of the females’ mates. By using a habituation-dishabituation paradigm, we found that unmated females could discriminate the urine scents of two male littermates from those of a novel male unrelated to the littermates. To understand how females use urinary cues to discriminate between males with different genetic relationships, we used gas chromatography coupled with mass spectrometry (GC-MS) to examine the volatile composition of urine from males with varying relatedness. It was found that KM male littermates shared similar volatile compositions in their urine. Our results suggest that male kinship reduces the Bruce effect in female KM mice, and provide additional evidence for mate choice being partly mediated by the Bruce effect in KM mice.

## Introduction

Early pregnancy in female mice (*Mus musculus*) may be terminated after the female is exposed to an unfamiliar male (Bruce effect) [1]. This phenomenon is common in many rodent species, both under laboratory conditions and in the wild [2], [3], [4]. Additional evidence for the Bruce effect is found in the domestic horse (*Equus ferus caballus*) [5] and in wild primates [6], [7]. The Bruce effect and its underlying mechanisms have been well-studied at both behavioral and neurobiological levels [8], [9], [10], [11]. The mechanism underlying pregnancy block in females includes two main processes, firstly the formation of a mating-induced memory of the stud male, and secondly the recognition of or discrimination between the stimulus male/odor and the stud male/odor. Newly mated female mice exposed to odors from the stud male and a syngeneic male show pregnancy block significantly less frequently than females exposed to odors from a novel male differing from the stud male in one gene (H-2K) [12]. This means that female mice can discriminate between chemosensory cues from the three groups of males and recognize the novel male’s odor. A wealth of literature shows that genetic relatedness and odors covary [13], [14], [15], and urinary chemical compounds differ significantly, both in composition and in the relative abundance of chemical compounds, between two strains of mice (Zhenjuan Men, unpublished data). However, whether a newly mated female can discriminate her mate’s male littermate from her mate via chemosensory cues in the urine based on the mating-induced memory, and whether exposure to the mate’s male littermate can elicit the Bruce effect, remains unclear. Are the chemical compositions in the chemosensory cues of the urine of male littermates similar or dissimilar?

The Bruce effect is considered to be a mate choice strategy, because aborting a pregnancy provides females with an opportunity to mate with males of higher quality [16]. It is also thought to be a strategy to reduce infanticide [17]. Mice are a territorial, polygynous, communally living species, and resident males aggressively defend their territories against intruders [18]. Young males usually disperse from their natal territory [19]. A newly inseminated female may be exposed to males that are genetically related to her mate, or to the scents of those males. Exposure to a male of a different genetic strain results in a higher incidence of pregnancy block than exposure to a male of the same genetic strain [20], [21], [22]. A wealth of literature has shown that volatile urinary constituents can code for individual identity in mice [23], [24], [25], [26]. Thus, the aims of our study are: 1) to test whether unmated female mice can discriminate between the scents of two male littermates and a novel male that is genetically unrelated to the littermates; 2) to test whether exposure of mated females to the urine scent from the male littermate of the females’ mate can lead to pregnancy block as predicted by the Bruce effect; 3) to test whether there are similarities in the chemical composition of urine scents from male littermate mice. From the perspective of mate choice and increasing inclusive fitness, we hypothesized that newly mated females should maintain their pregnancies if they are exposed to chemosensory cues from their mate’s male littermates. That is to say, the incidence of pregnancy block in females exposed to urine scents from male littermates of their mates should be similar to that of females exposed to the scents of the stud male, and lower than that of females exposed to the scents of novel males. To test this hypothesis, we first evaluated whether unmated females could discriminate among the urine scents of a male, the male’s male littermate, and a novel male unrelated to both, by applying the paradigm of habituation-dishabituation. Then, we measured the miscarriage rate in females exposed to urine scents from males that were either littermates of their mates or unrelated to their mates. Lastly, we analyzed the urinary volatile composition of males of each group using gas chromatography and mass spectrometry (GC-MS).

## Materials and Methods

### Animals and Ethics Statement

Sexually naive female Kunming (KM) mice were purchased from the Weitong-Lihua Experimental Animal Company, Beijing, China at 10 weeks of age, and were acclimatized for 2 weeks prior to use. Sixteen male KM mice from three different litters (denoted “A” (4 males), “B” (5 males) and “C” (7 males)) were purchased from the Laboratory Animal Center of Academy of Military Medical Sciences, Beijing, China. These males were used as mates and their male littermates. Six additional male KM mice, unrelated to the mate/littermate males, were purchased from the Weitong-Lihua Experimental Animal Company, Beijing, China and used as novel males (denoted “W”).

Mice were individually housed in plastic cages (28 × 18 × 16 cm) under a reversed 12L: 12D light/dark photoperiod (lights on at 2100 h) inside an animal housing room (biosafety level 2 laboratory) [27]. Food and water were provided *ad libitum*. Wood shavings were used as bedding and were changed once a week. The room was maintained at 20±0.5°C and ventilated continuously. To ameliorate suffering before sacrificing the subjects in a separate operating room away from the animal housing room, we used an overdose anesthesia (30% ethyl carbamate, 1.5 ml/100 g) injection. The use, management and welfare of the study animals met the Chinese Animal Care Regulations for Captive and Laboratory Animals as outlined in the 1988 National Regulation on Laboratory Animal Research, issued by the Ministry of Science and Technology. Our protocols were approved by the Institutional Animal Care and Use Committee of Beijing Normal University.

### Urine Collection

To allow urine to be collected, each donor male was placed in a clean cage and allowed to acclimatize for 3 min. When urination took place, urine was immediately collected with a pipette and transferred to a 0.5 ml centrifuge tube. Urine samples collected on different days from the same donor male were mixed together for the behavioral tests and the GC-MS analyses. The urine samples were stored at −20°C and defrosted at room temperature prior to use. To extract urinary compounds, 300 µl dichloromethane was added to a vial containing 300 µl of urine. The mixture was then stored at room temperature for 2 h, maintained at 4°C for 12 h and then transferred into another vial for chemical analysis.

### Discrimination between Related and Unrelated Males

Before mating, we first used a habituation-dishabituation paradigm to determine whether females could distinguish the urine scents of males from the same litters. This protocol has been used in several previous studies [28], [29]. In brief, subject females were initially and individually tested in 4 consecutive habituation trials, in which they were exposed to the urine scent of an individual male each time, followed by a test trial using the urine scent from the male’s male littermate. In the habituation trials, each subject was exposed to both urine and water (as a control); each trial lasted 3 min and a 2-min interval occurred between consecutive trials. For the test trial, the urine of the familiar male was replaced by the urine of his male littermate. We injected 4 µl of urine into a disposable glass capillary (1.1 mm in diameter, 12 cm in length) and manually (wearing disposable gloves) presented the sample-containing tip to the female in her cage. The total time that the female spent sniffing the urine was recorded. Subsequently, we also used a cross-habituation dishabituation paradigm to test whether female mice habituated to the urine of one male could discriminate between his male littermate and a novel, unrelated male by urine scents. The habituation-dishabituation paradigm is used to detect the female’s ability to distinguish the two urine odors. The cross habituation-dishabituation paradigm is used to test the female’s ability to distinguish the degree of similarity among three urine odors. Details of the cross-habituation discrimination method are provided in Johnston’s and Liu’s studies [30], [31]. In short, four habituation trials were conducted as in the previous experiment. We then simultaneously presented the female with the urine of the original male’s male littermate and that of a novel, unrelated male, and recorded the total time that the female spent sniffing each. All behavioral tests were performed during the dark phase.

### Pregnancy Block Test

Three weeks after the habituation-dishabituation tests, we used the same females for the pregnancy block test. Each female was paired with a male at 0900 h and then checked at 1100, 1500 and 1900 h for the presence of a vaginal plug, which was evidence of successful mating. To ensure the retention of familiarity, mated females remained with their mates until 2100 h, before being transferred to clean individual cages. Mated females were randomly assigned to male urine scent groups: mates (Group 1, n = 15), mates’ male littermates (Group 2, n = 15), and unrelated males (Group 3, n = 21).

Individual females were briefly restrained by scruffing, and 40 µl of urine was placed on their oronasal groove. Urine exposures were conducted twice daily, at 0800 and 2000 h, on the 3 consecutive days following mating. Eight days after mating, females were sacrificed and their uteri were examined for implantation sites.

### Analysis of Urine Volatiles

Analytical GC-MS (gas chromatography coupled with mass spectrometry) was performed on a 320 GC/MS/MS instrument (Burker Company, US) with the library NIST 2002 (National Institute of Standards and Technology database). An MS workstation (Windows XP) was used for data acquisition and processing. The GC was equipped with a 30-m glass capillary column (VF-5, internal diameter 0.25 mm×0.25 µm film). Helium was used as the carrier gas at a flow rate of 1.0 ml/min. The temperature of the injector was set at 250°C. The oven temperature was programmed as follows: 50°C as the initial temperature, increased by 5°C /min to 150°C and then by 10°C /min to 230°C, maintained for 2 min, and then increased by 30°C /min to 290°C and held for 5 min. Finally, the temperature was maintained at 290°C for 5 min post-run to clean the column. Electron impact ionization was used at 70 eV. Scanning mass ranged from 30 to 600 amu. Urine samples were injected in 1 µl volumes in splitless mode [23].

Chemical compound identification was undertaken by comparing the mass spectra of GC peaks with those of the Mass Selective Detector using the NIST 2002 library. Compounds absent from the NIST database were identified via comparison with chemical spectra in the literature [23].

### Statistical Analysis Procedure

For the habituation-dishabituation and cross-habituation dishabituation tests, we first examined the raw data using the Kolmogorov-Smirnov test, which revealed that all data were normally distributed and therefore met model assumptions for the subsequent paired *t* tests. Differences in pregnancy failure rates among the three treatments were tested for by using Fisher’s exact probability tests. First, we tested the pregnancy block data together by 2×3 contingency table followed by the Fisher exact test for each two groups by 2×2 contingency table. We conducted the Cochran-Armitate trend test to evaluate whether the incidence of pregnancy block was correlated with male familiarity and identity coded by urine scent, which depends on genetic relatedness. We expected that the more closely genetically related the males, the lower the incidence of pregnancy abortion. For the GC-MS data, we used the method described by Zhang *et al.* to quantify the absolute and relative abundances of relevant compounds in litters [23]. We used the total peak area of each compound as the measure of absolute abundance, and then divided that number by the sum of the peak areas of all 11 GC peaks. We then multiplied that value by 100 to obtain the percentage relative abundance. We first used Principal Component Analysis (PCA) to classify the relative abundances of the 11 compounds in males of the 4 groups (from litters A, B and C, and males unrelated to them). Simple scatter plots were then drawn based on different combinations of the 4 extracted principal components [31]. We also used the RSD (relative standard deviation) to describe variation in components within and between different groups: RSD = SD/Mean×100. SD is the standard deviation of individuals, and the mean was the average of each volatile component’s relative abundance across individuals. A bigger RSD value indicates a bigger variance between two groups.

The Cochran-Armitate trend test was performed using SAS statistical software, version 9.2 (SAS Institute Inc., Cary, NC). Other statistical analyses were conducted using SPSS, version 16 (SPSS Inc., Chicago, US). All tests except the Fisher’s exact probability tests were two-tailed. We used one-tailed tests to test for differences in pregnancy block rate between females exposed to the scent of their mate’s male littermate and those exposed to the scent of a novel male, because our prediction was directional. The level of significance was set at α = 0.05.

## Results

### Female Mice can Discriminate between male Littermates

**Figure 1:**
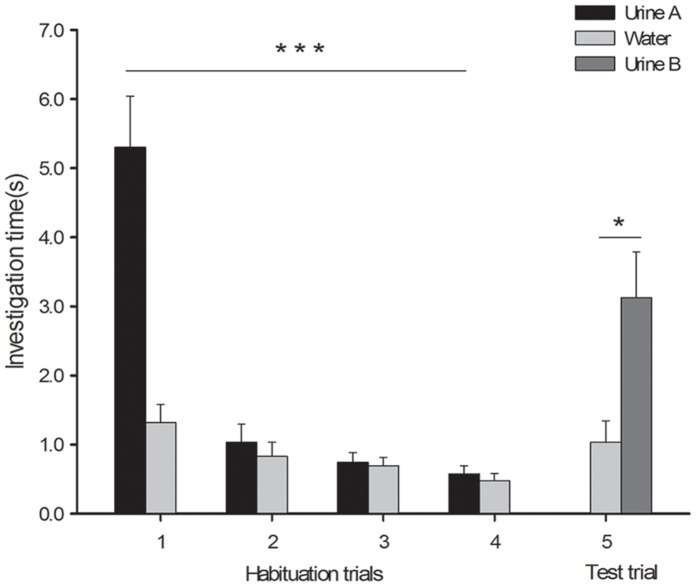
Time spent by females investigating the urine of two different donor males. Urine A: urine of male A; Urine B: urine of male A’s male littermate (male B). Females were habituated to the urine of one male and then tested in a dishabituation trial with the urine of the original male’s male littermate (mean ± SE, n = 10; ***: p<0.001, *: p<0.05).

In the first habituation trial of the habituation-dishabituation experiment, females spent significantly more time sniffing the scent of male urine than sniffing the control (water) (5.30±0.74 vs. 1.32±0.26 s, t = 5.496, p = 0.0001, n = 10; Figure 1); however, by trial 4, the amount of time spent sniffing the male scent had decreased significantly (trial 1 vs. trial 4: 5.30±0.74 s vs. 0.58±0.12 s, t = 6.146, p = 0.0001, n = 10), demonstrating the habituation of the females to it. In the test trial, females spent more time sniffing the urine of the males’ male littermates (Figure 1) than that of the control (3.13±0.67 s vs. 1.32±0.26 s, t = 2.465, p = 0.036, n = 10).

**Figure 2:**
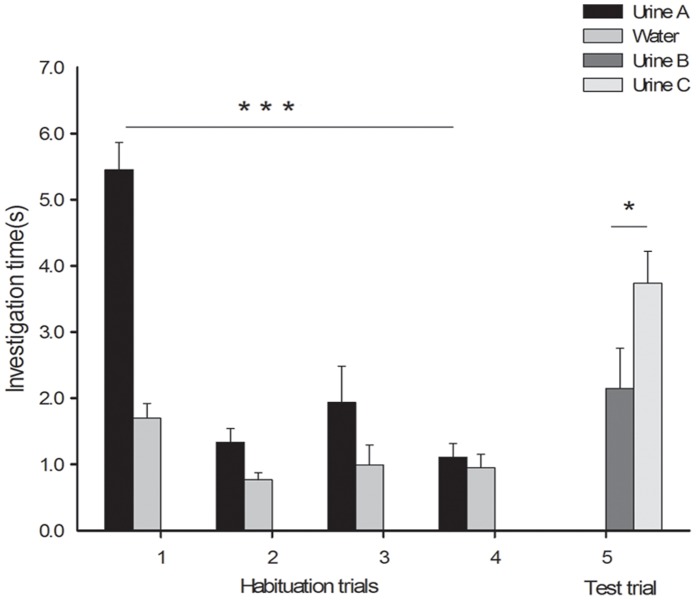
Time spent by females investigating the urine of three different donor males. Urine A: urine of male A; Urine B: urine of male A’s male littermate (male B); Urine C: urine of a male unrelated to males A and B. Females were habituated to the urine of one male and then tested in a dishabituation trial with the urine of the familiar male’s male littermate and a male unrelated to both other males (mean ± SE, n = 13; ***: p<0.001, *: p<0.05).

As shown in Figure 2, in their first habituation trials, females in the cross-habituation discrimination experiment showed more interest in the urine stimulus than in the control (5.45±0.41 s vs. 1.69±0.23 s, t = 8.091, p = 0.0001, n = 13). As with the previous experiment, habituation occurred by trial 4 (trial 1 vs. trial 4: 5.45±0.41 vs. 1.11±0.21 s, t = 8.873, p = 0.0001, n = 13). In the test trial, females spent significantly more time sniffing the urine of unrelated males than that of their mates’ male littermates (3.74±0.48 s vs. 2.15±0.61 s, t = 2.451, p = 0.031, n = 13).

### Male Kinship Reduces the Bruce Effect in Female Mice

The incidence of pregnancy failure was higher in females exposed to the urine scent of males unrelated to their mates (p = 0.033, 2×3 contingency table). Exposure to the urine of a novel male resulted in pregnancy failure in 9 out of 21 mice (43%), whereas exposure to the mate’s urine resulted in pregnancy failure in 1 out of 15 mice (7%), a significant difference (p = 0.019, one-tailed test). Only 2 of 15 mice (13%) terminated their pregnancy when exposed to urine from their mate’s male littermate. This pregnancy failure rate was similar to that of females exposed to their mate’s urine (p* = *0.500, one-tailed test), and did not differ from that of females exposed to novel males’ urine scent (p = 0.061, Table 1). The Cochran-Armitage trend test showed that male familiarity/similarity was significantly and negatively associated with pregnancy block (p = 0.0089): females exposed to urine scents from novel males are more likely to experience pregnancy block.

**Table 1:** The effectiveness of male urine in inducing pregnancy block in newly mated female mice.

Treatment exposure to	Number of females showing pregnancy failure	Number of females showing pregnancy	Pregnancy failure (%)
Mate’s urine	1	14	7^a^
Mate’s male littermate’s urine	2	13	13^ab^
Novel male’s urine	8	12	43^b^

Groups with shared letters do not differ from each other statistically.

### Littermates Share Similar Urinary Compounds

**Figure 3:**
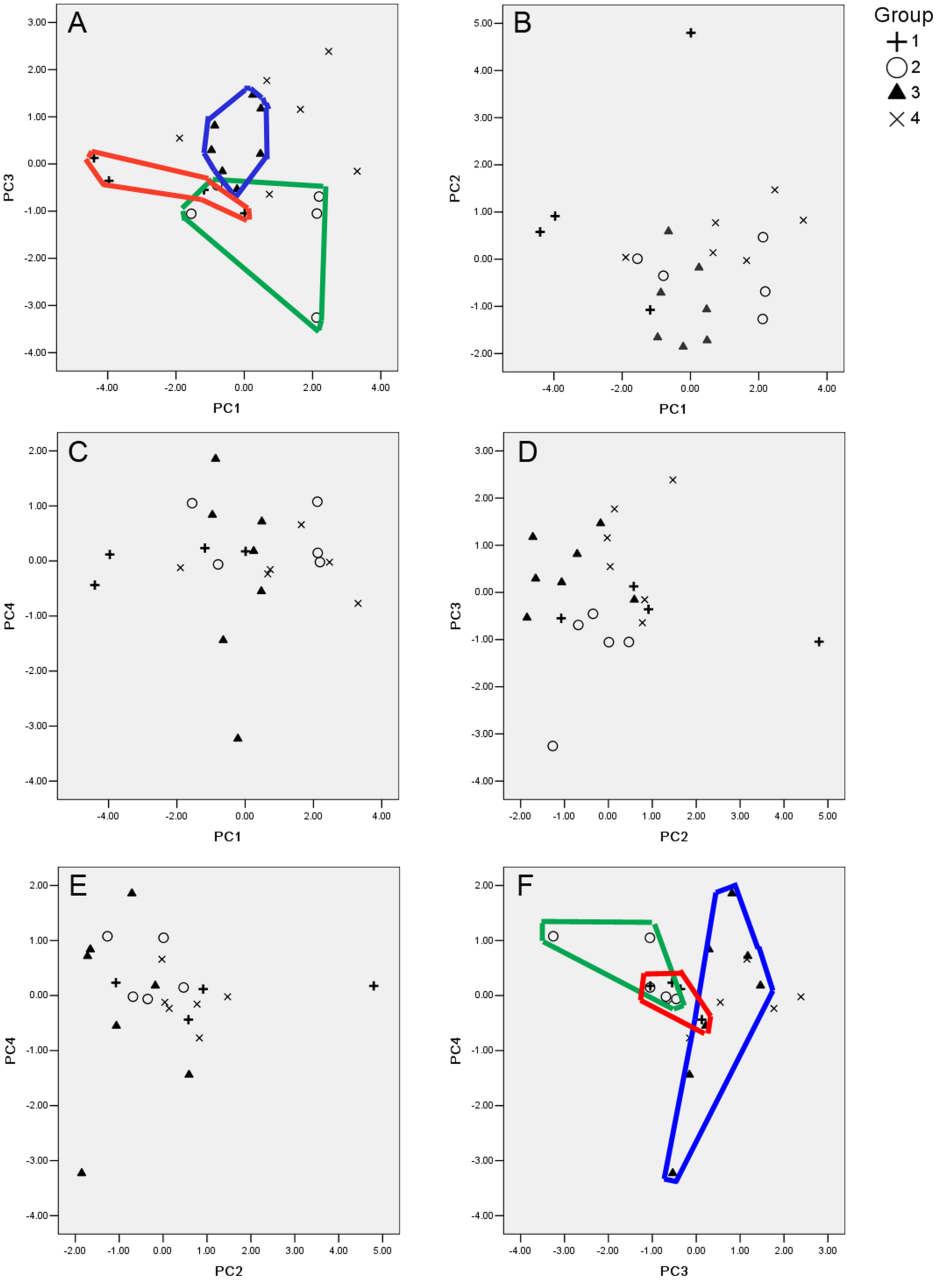
Scatter plots based on the results of PCA. (a) PC_1_ versus PC_3_; (b) PC_1_ versus PC_2_; (c) PC_1_ versus PC_4_; (d) PC_2_ versus PC_3_; (e) PC_2_ versus PC_4_; (f) PC_3_ versus PC_4_. Group 1: litter A, n = 4; group 2: litter B, n = 5; group 3: litter C, n = 7; group 4: individuals unrelated to the males in the other groups, n = 6. Each solid line encircling the plots encloses individuals from the same litter.

We putatively identified eleven urinary volatile compounds by GC-MS in KM male urine (Table 2). PCA of the relative abundance of the 11 chemical compounds extracted 4 principal components (PCs). The percentage variance explained by PC_1_ to PC_4_ was 34.6, 18.3, 13.3, and 9.1%, respectively, together accounting for 75.3% of the total variance. According to the rotated component loadings, we found that PC_1_ was most related to component (S)-2-sec-buryl-4,5-dihydrothiazole, PC_2_ to R,R-3,4-dehydro-exo-brevicomin, PC_3_ to o-toluidine, and PC_4_ to phenol,4-ethyl (Table 3). The scatter plots grouped individuals from the same litter together based on PC_1_ versus PC_3_ and PC_3_ versus PC_4_ (Figure 3 A, F). No such grouping resulted from the other combinations of PCs (Figure 3 B, C, D, E). We also found that within-litter RSDs were smaller than the among-litter RSD (Table 2). These results indicate that genetically related KM male mice share similar profiles and patterns in relative abundances of urinary volatile components.

**Table 2:** Relative abundance of volatiles in the urine of male KM mice (mean ± SD), and individual variation (RSD) in their relative abundance in mice from different litters.

Peak number	Retention time(min)	Compounds	Relative abundance				
			A (n = 4)	B (n = 5)	C (n = 7)	W (n = 6)	All (n = 22)
1	5.17	Unknown	10.2±12.1	23.5±6.2	17.5±3.5	21.7±7.1	18.6±8.1
2	7.40	Unknown	2.6±3.4	8.4±3.3	6.6±1.7	7.3±3.4	6.5±3.3
3	8.00	2-Heptanone	0	0.13±0.3	0	0	0.03±0.1
4	8.15	5-Hepten-2-one	0	0.1±0.1	0.9±0.8	0.8±0.5	0.54±0.6
5	8.62	Unknown	0	0.8±0.5	0.6±0.4	1.2±0.7	0.71±0.6
6	8.94	Dimethyl sulfone	13.5±6.9	6.54±2.0	6.2±1.9	10.2±2.4	8.7±4.3
7	9.48	6-Hydroxy-6-methyl-3-heptanone and5,5-dimethyl-ethyltereahydrofuran-2-ol	4.5±4.1	6.9±3.3	5.4±2.1	9.6±4.6	6.74±3.8
8	13.62	R,R-3,4-dehydro-exo-brevicomin	24.6±8.6	18.8±3.8	14.1±5.6	14.6±4.1	17.2±6.5
9	14.35	o-Toluidine	1.2±0.7	0.2±0.2	0.7±0.6	1.3±1.0	0.8±0.8
10	15.49	(S)-2-sec-buryl-4,5-dihydrothiazole	37.9±25.3	33.3±8.2	37.8±8.2	31.7±8.2	35.1±12.1
11	17.34	Phenol,4-ethyl	5.40±4.3	1.4±1.9	6.3±6.2	1.5±3.5	3.7±4.8
RSD			57.7	72.9	47.9	58.5	102.6

A, B, C: individuals from litters A, B and C, respectively, purchased from the Laboratory Animal Center of Academy of Military Medical Sciences. W: individuals of unknown relationship (but unrelated to A, B, or C males) purchased from the Weitong-Lihua Experimental Animal Company.

RSD: Relative Standard Deviation.

**Table 3:** Component loadings for the first 4 PCs of the GC-MS peak areas in urine.

Compounds	Rotated component loadings			
	PC_1_	PC_2_	PC_3_	PC_4_
Unknown	0.775	0.279	−0.247	0.413
Unknown	0.778	0.388	−0.221	0.276
2-Heptanone	0.187	−0.010	−0.717	0.095
5-Hepten-2-one	−0.178	0.427	0.329	0.642
Unknown	0.616	0.550	0.285	0.099
Dimethyl sulfone	0.096	−0.734	0.328	−0.149
6-Hydroxy-6-methyl-3-heptanone and 5,5-dimethyl-ethyltereahydrofuran-2-ol	0.844	0.050	0.008	0.004
*R,R*-3,4-dehydro-exo-brevicomin	−0.208	−0.830	0.041	0.114
o-Toluidine	0.185	−0.246	0.783	0.164
(S)-2-*sec*-buryl-4,5-dihydrothiazole	−0.857	0.307	−0.079	0.037
Phenol,4-ethyl	−0.320	0.167	0.097	−0.758
% Variance	34.633	18.283	13.261	9.099

PC: Principal Component.

## Discussion

We explored the Bruce effect in mice by applying both behavioral tests and chemical analysis of the urine to females, male littermates, and unrelated males. We found that the pregnancy block rate was significantly lower in recently mated KM female mice that were exposed to the urine scent of their mates, than in females exposed to the scent of unrelated males. However, the abortion rate was similar in females exposed to urine scents from their mates and in those exposed to the scent of their mates’ male littermates. In line with this result, the female’s mate and his male littermates share a similar urinary volatile components profile, but male littermates and unrelated males have different profiles and a larger RSD. The behavioral tests, however, indicate that unmated females can distinguish the urine scents of two male littermates from those of two unrelated males.

Urine scent contains cues conveying information on individual identity, social status, gender and reproductive status in many mammal species [23], [32], [33], [34], and urine contains different chemical compounds present at different ratios, so that it is ideal for coding individuality in mammals and birds [31], [32], [35]. The urine odor is also covariant with genes in *Mus* species [14]. The unmated female mice in our study could distinguish the minor difference in the scents of two male littermates, yet recently mated females showed similar pregnancy block rates after being exposed to urine scents from male littermates. This result with regards to pregnancy block is consistent with the results of Yamazaki et al. [12], who exposed females to stud and syngeneic males. On one hand, this result may indicate that the mating process influences females’ discriminative ability. Mating causes significant changes in parts of the female’s brain which may regulate this ability [36]. On the other hand, individual discrimination, as shown by the habituation-dishabituation paradigm and the cross habituation-dishabituation paradigm, is an easy task for female mice [37], [38]. The female may simply treat the second scent as different if she can detect a minor difference in the chemosensory cue. Individual discrimination in the Bruce effect, however, may be a more difficult task for the female, because she needs to retrieve the memory of her mate’s scent and compare it with the new scent of the stimulus male to achieve individual recognition. The similarity in both the chemical compounds and their relative abundances in urine from male littermates may provide additional evidence for this individual memory and recognition process. Females might learn to use the mate’s scent as a template to decide to abort a pregnancy or not, based on the degree of similarity between the stimulus male’s scent and their mate’s scent. Empirical studies have shown that female mice make the decision to terminate the pregnancy or not based on chemosignal similarities [39]. The large RSD and dissimilarity in chemical composition and relative abundances between the urine scents from the female’s mate and the novel male might explain the differences we found in pregnancy block rates in the recently mated female mice (Table 1, 2; Figure 3). The kinship between the female’s mate and his male littermate reduced the pregnancy block rate in the female mice. Moreover, males show a high degree of tolerance towards their brothers [40]. Mate sharing is common, and fighting is less frequent between brothers than between unrelated males [41]. In this case, if a female can assess the kinship of males, she has no need to terminate her current pregnancy if she meets her mate’s brother, which may happen frequently in gregarious communities. This may help to increase the fitness of both female and male mice.

Our results are inconsistent with those from an early study in which inseminated females were exposed to their mates’ male littermates: the pregnancy failure rate caused by exposure to the mate was 24%, while the pregnancy failure rate caused by the mate’s male littermates was 42% [42]. In addition to differences in the genetic relationship of the stimulus males with the females, differences in animal strain may explain the differences between the findings in these two studies [43], [44]. We used KM mice, while Bloch and Wyss used NMRI mice. Further comparisons of the genetic backgrounds, urinary compositions and behaviors of mice from these two strains may clarify this issue. Lastly, the different experimental methods used (direct body contact vs. indirect contact via urine scent) may also contribute to the differences between the results of the two studies.

When the Bruce effect occurs through indirect contact via urine scent, one or more urinary compounds may play a key role in conveying information on the individual identity of the male urine donors. MUPs and their bound testosterone-dependent ligands are proposed to convey maleness and individuality in the pregnancy block effect [9]. We found the same compounds (3,4-dehydro-exo-brevicomin and 2-sec-butyl-4,5-dihydrothiazole) in our GC-MS results as were found in previous studies [45], [46]. However, these volatile compounds have not been found to induce pregnancy block but to accelerate puberty [45], [46]. Therefore, the possibility of a combined effect of MUPs and urinary volatile compounds on the Bruce effect cannot be excluded. Mate recognition and pregnancy block have been found to be mediated by MUPs and associated volatile chemicals acting upon the vomeronasal organ and either the accessory olfactory bulb or the main olfactory bulb [47].

MUPs have been proposed as playing a role in conveying individuality in previous studies. Our study was limited by our inability to control for the role of MUPs in our experimental design, due to the close association between MUPs and urinary volatile constituents. A further experiment analyzing similarities in MUPs in the urine of male littermates would be needed to separate out more fully the causes of pregnancy block in female mice.

In summary, kin relationships minimize the Bruce effect in this out-bred strain of KM mice. Variation in urinary volatile compound composition and relative abundance may promote the ability of females to discriminate between previous mates and novel males. The ability of females to discriminate males based on levels of kinship may help this species to maintain communal life. This study not only enhances our understanding of the communal social life of KM mice, but also that of other rodent species and mammals in which the Bruce effect can occur.
